# Supplementary material for: Reversal of High-Fat Diet-Induced Non-Alcoholic Fatty Liver Disease by Metformin Combined with PGG, an Inducer of Glycine N-Methyltransferase
Source: Int J Mol Sci. 2022 Sep 3;23(17):10072. doi: 10.3390/ijms231710072 (PMC9456083; doi:10.3390/ijms231710072)
Supplement: Supplementary file 1 [file ijms-23-10072-s001.zip › ijms-1864021-supplementary.pdf]

Supplementary material

Reversal of High-Fat Diet-induced Non-Alcoholic Fatty Liver Disease by Metformin Combined with PGG, an inducer of Glycine N-methyltransferase

Ming-Hui Yang, Wei-You Li, Ching-Fen Wu, Yi-Ching Lee, Allan Yi-Nan Chen, Yu-Chang Tyan*, Yi-Ming Arthur Chen*

Table of contents

Supplementary Figure S1 ……..…………………………………..…..……Page 2

Supplementary Table S1. ……………………………..…………..…..…Page 3

Supplementary Table S2. ……………………………..…….……..……Page 3

Figure

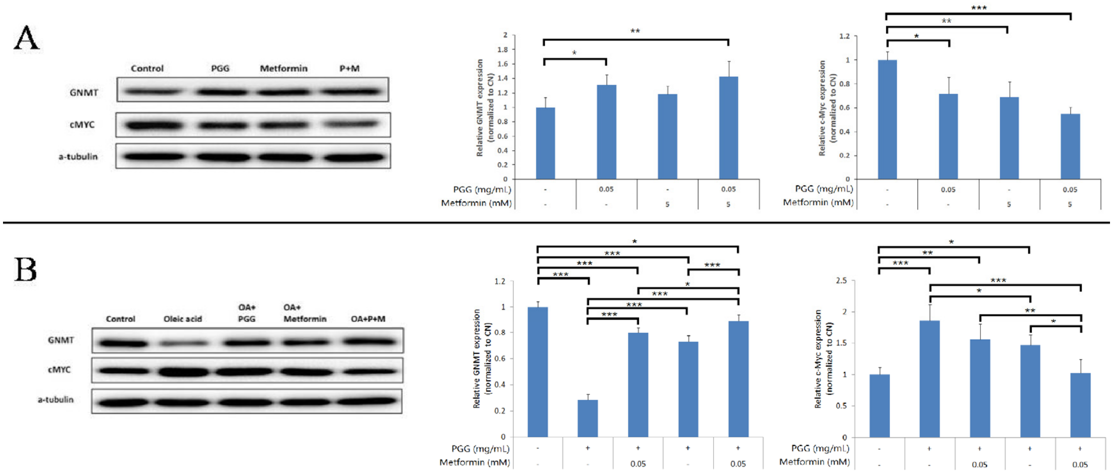

Supplementary Figure S1. Protein expression detection of GNMT and c-Myc from Mahlavu cells without oleic acid (A) and with oleic acid(B). (*$p<0.05$;**$p<0.01$; ***$p<0.001$) (N=3/group)

Tables

Supplementary Table S1. Primer sequences.

Target	Primer sequences (5'-3')
GNMT	Forward: GTTGACGCTGGACAAAGA
	Reverse: AGCCTGTGCTGAGGATA
SREBP-1c	Forward: CACTTCTGGAGACATCGCAAAC
	Reverse: ATGGTAGACAACAGCCGCATC
PPARα	Forward: CAAGGCCTCAGGGTACCACT
	Reverse: TTGCAGCTCCGATCACACTT
PPARγ	Forward: ATTCTGGCCCACCAACTTCGG
	Reverse: TGGAAGCCTGATGCTTTATCCCCA
TNFα	Forward: GCCTCTTCTCATTCCTGCTTG
	Reverse: CTGATGAGAGGGAGGCCATT
IL-1β	Forward: CCAGCTTCAAATCTCACAGCAG
	Reverse: CTTCTTTGGGTATTGCTTGGGATC
β-actin	Forward: TGCTCGGGACGTTCACAAC
	Reverse: GAGAATAAAGCAACTGCACAAACAA

Supplementary Table S2. The steatosis scores among different groups of mice.

Group	Score
Control	0
HDF	2.29 ± 0.27
HDF + metformin	1.38 ± 0.42
HDF + PGG	2.13 ± 0.29
HDF + metformin + PGG	1.13 ± 0.4 *

*: p<0.05 ; (N=10/group)
